# Cholesterol 25-hydroxylase suppresses SARS-CoV-2 replication by blocking membrane fusion

**DOI:** 10.1073/pnas.2012197117

**Published:** 2020-11-25

**Authors:** Ruochen Zang, James Brett Case, Eylan Yutuc, Xiucui Ma, Sheng Shen, Maria Florencia Gomez Castro, Zhuoming Liu, Qiru Zeng, Haiyan Zhao, Juhee Son, Paul W. Rothlauf, Alex J. B. Kreutzberger, Gaopeng Hou, Hu Zhang, Sayantan Bose, Xin Wang, Michael D. Vahey, Kartik Mani, William J. Griffiths, Tom Kirchhausen, Daved H. Fremont, Haitao Guo, Abhinav Diwan, Yuqin Wang, Michael S. Diamond, Sean P. J. Whelan, Siyuan Ding

**Affiliations:** ^a^Department of Molecular Microbiology, Washington University School of Medicine in St. Louis, St. Louis, MO 63110;; ^b^Key Laboratory of Marine Drugs, Ministry of Education, Ocean University of China, 266100 Qingdao, China;; ^c^Department of Medicine, Division of Infectious Diseases, Washington University School of Medicine in St. Louis, St. Louis, MO 63110;; ^d^Swansea University Medical School, SA2 8PP Swansea, United Kingdom;; ^e^Center for Cardiovascular Research and Division of Cardiology, Department of Internal Medicine, Washington University School of Medicine in St. Louis, St. Louis, MO 63111;; ^f^John Cochran VA Medical Center, St. Louis, MO 63106;; ^g^Department of Microbiology and Immunology, Indiana University School of Medicine, Indianapolis, IN 46202;; ^h^Department of Pathology and Immunology, Washington University School of Medicine in St. Louis, St. Louis, MO 63110;; ^i^Program in Molecular Cell Biology, Washington University School of Medicine, St. Louis, MO 63110;; ^j^Program in Virology, Harvard Medical School, Boston, MA 02115;; ^k^Program in Cellular and Molecular Medicine, Boston Children’s Hospital, Boston, MA 02115;; ^l^Department of Cell Biology, Harvard Medical School, Boston, MA 02115;; ^m^Autonomous Therapeutics, Inc., New York, NY 10013;; ^n^Department of Biomedical Engineering, McKelvey School of Engineering, Washington University in St. Louis, St. Louis, MO 63110

**Keywords:** SARS-CoV-2, interferon, virus entry, COVID-19, innate immunity

## Abstract

Cholesterol 25-hydroxylase (*CH25H*) is an interferon (IFN)-stimulated gene that shows broad antiviral activities against a wide range of enveloped viruses. Here, using an IFN-stimulated gene screen against vesicular stomatitis virus (VSV)-SARS-CoV and VSV-SARS-CoV-2 chimeric viruses, we identified CH25H and its enzymatic product 25-hydroxycholesterol (25HC) as potent inhibitors of SARS-CoV-2 replication. Internalized 25HC accumulates in the late endosomes and potentially restricts SARS-CoV-2 spike protein catalyzed membrane fusion via blockade of cholesterol export. Our results highlight one of the possible antiviral mechanisms of 25HC and provide the molecular basis for its therapeutic development.

## Significance

The novel severe acute respiratory syndrome coronavirus-2 (SARS-CoV-2), the etiological agent of coronavirus disease-2019 (COVID-19), has swept the world in unprecedented speed. In a few months, SARS-CoV-2 has infected millions of people and caused tens of thousands of deaths. There are no Food and Drug Administration-approved antivirals or vaccines yet available and clinical treatments are limited to supportive therapies that help alleviate the symptoms. Thus, there is an urgent need to identify effective antivirals as countermeasures before safe and effective vaccines are developed, tested, and then produced on a large scale. Our approach is to harness the germline-encoded interferon antiviral response to inhibit SARS-CoV-2 replication thereby limiting its pathogenicity.

The novel severe acute respiratory syndrome coronavirus-2 (SARS-CoV-2), the etiological agent of coronavirus disease-2019 (COVID-19) (1, 2), has infected millions of people worldwide and caused hundreds of thousands of deaths. Currently, there are no Food and Drug Administration-approved vaccines available. In most instances, treatment is limited to supportive therapies to help alleviate symptoms. With the concern that monotherapy would rapidly result in the emergence of resistance, there is a pressing need to identify multiple effective antivirals as countermeasures before safe and efficacious vaccines are developed and deployed. Here, we sought to harness the host innate immune responses to inhibit SARS-CoV-2 replication. Interferons (IFNs) are a group of small, secreted proteins (3, 4) that potently suppress the replication of many viruses through the action of hundreds of IFN-stimulated genes (ISGs) (5). IFN and ISG levels are up-regulated in SARS-CoV-2–infected cells and lung tissues from COVID-19 patients (6, 7). Compared to SARS-CoV, SARS-CoV-2 appears to be more sensitive to the antiviral activities of IFNs (8). SARS-CoV-2 replication is inhibited by IFN treatment in both immortalized and primary cells (9–11). While direct IFN administration often results in adverse effects in humans (12, 13), a targeted approach of activating the antiviral effects of specific ISGs holds promise.

To identify potential ISG effector proteins that act to block coronavirus (CoV) at the entry or egress stages of the replication cycle, we utilized replication-competent chimeric vesicular stomatitis virus (VSV) eGFP reporter viruses decorated with either full-length SARS-CoV spike (S) protein or SARS-CoV-2 S in place of the native glycoprotein (G) (14). We used an HEK293 cell line that stably expresses plasma membrane-localized mCherry-tagged human ACE2, the SARS-CoV and SARS-CoV-2 receptor (2, 15–17) (*SI Appendix*, Fig. S1*A*). HEK293-hACE2-mCherry cells supported 100-fold more VSV-SARS-CoV-2 replication than wild-type HEK293 cells (*SI Appendix*, Fig. S1 *B*–*D*). We recently showed robust SARS-CoV-2 infection of primary human intestinal enteroids (18). By RNA-sequencing of these intestinal enteroid cultures, we identified the ISGs most highly and commonly induced by type I IFN (IFN-β) and type III IFN (IFN-λ). We transduced HEK293-hACE2 stable cells with lentiviruses encoding 57 of these individual ISGs and tested their ability to suppress VSV-SARS-CoV and VSV-SARS-CoV-2 replication.

**Fig. 1.**
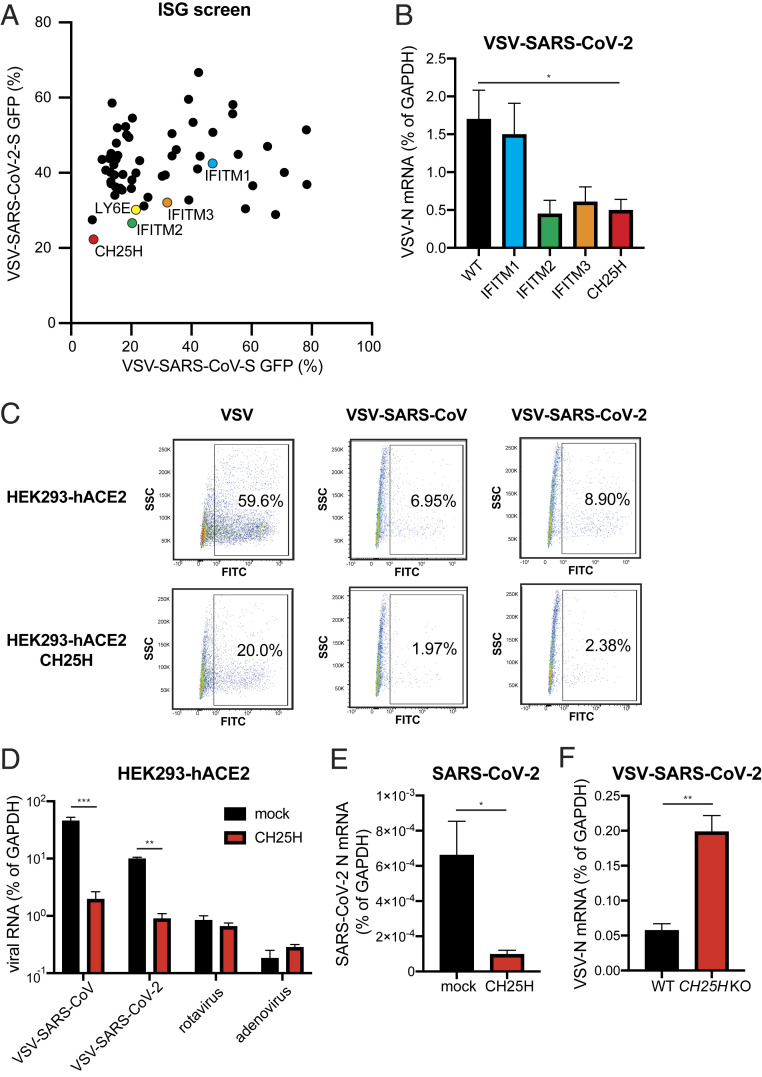
ISG screen identifies CH25H as an antiviral host factor that restricts SARS-CoV-2 infection. (*A*) HEK293-hACE2-mCherry cells were transduced with lentiviral vectors encoding individual ISGs for 72 h and infected with VSV-SARS-CoV or VSV-SARS-CoV-2 (MOI = 1) for 24 h. The percentage of GFP^+^ cells were quantified and plotted. Five ISGs were colored and selected for further analysis. (*B*) Wild-type (WT) HEK293-hACE2 cells or HEK293-hACE2 cells stably expressing indicated ISGs were infected with VSV-SARS-CoV-2 (MOI = 1). At 18 hpi, the mRNA level of VSV N was measured by qRT-PCR and normalized to GAPDH expression. (*C*) HEK293-hACE2 cells with or without CH25H expression were infected with wild-type VSV, VSV-SARS-CoV, or VSV-SARS-CoV-2 (MOI = 10) for 6 h. Cells were harvested and measured for GFP percentage and intensity by flow cytometry. Numbers indicate the percentage of FITC^+^ virus-infected cells within the entire population. (*D*) HEK293-hACE2 cells with or without CH25H expression were infected with VSV-SARS-CoV, VSV-SARS-CoV-2, rhesus rotavirus RRV strain, or adenovirus serotype 5 (MOI = 3) for 24 h. The levels of VSV N, rotavirus NSP5, and adenovirus hexon were measured by qRT-PCR and normalized to GAPDH expression. (*E*) HEK293-hACE2 cells with or without CH25H expression were infected with a clinical isolate of SARS-CoV-2 (2019-nCoV/USA-WA1/2020 strain, MOI = 0.5). At 24 hpi, the mRNA level of SARS-CoV-2 N was measured by qRT-PCR and normalized to GAPDH expression. (*F*) HEK293-hACE2 cells expressing CH25H were transduced with mock or CH25H CRISPR/Cas9 targeting lentiviruses and infected with VSV-SARS-CoV-2 (MOI = 1). At 24 hpi, the mRNA level of VSV N was measured by qRT-PCR and normalized to GAPDH expression. For all panels except *A*, experiments were repeated at least three times with similar results. The experiment in *A* was performed twice with average numbers indicated on the graph. Raw data are listed in Dataset S1. Data are represented as mean ± SEM. Statistical significance is from pooled data of the multiple independent experiments (*P ≤ 0.05; **P ≤ 0.01; ***P ≤ 0.001).

Ectopic expression of AXIN2, CH25H, EPSTI1, GBP5, IFIH1, IFN-induced transmembrane protein (IFITM)-2, IFITM3, and LY6E resulted in a marked reduction (<36%) in the infectivity of both viruses, indicated by the number of GFP-infected cells (Fig. 1*A* and Dataset S1). Among these genes, *IFIH1* (which encodes MDA5) activates IFN signaling upon ectopic expression (19). LY6E and IFITMs recently were reported to inhibit SARS-CoV-2 (20–22) and thus served as positive controls for our assay. We validated the top candidates in HEK293-hACE2 cells expressing CH25H, IFITM1, IFITM2, or IFITM3 (*SI Appendix*, Fig. S1*E*). Consistent with our screen results, the expression of IFITM2 or IFITM3 but not IFITM1 suppressed VSV-SARS-CoV-2 infection, as evident by a reduction in viral mRNA and protein levels (Fig. 1*B* and *SI Appendix*, Fig. S1*F*). CH25H was comparable to IFITM2 and blocked virus replication at 18 h postinfection (hpi) (Fig. 1*B*).

*CH25H* encodes cholesterol 25-hydroxylase that catalyzes the formation of 25-hydroxycholesterol (25HC) from cholesterol (23). In many cell types, 25HC is further converted to 7-α, 25-dihydroxycholesterol (7-α, 25-diHC), an oxysterol that functions as a chemoattractant for T cells and B cells (24). 25HC exhibits broad inhibitory activities against enveloped viruses of different families (25, 26), including two porcine CoVs (27). Within a single-cycle of replication (6 hpi), CH25H expression slightly inhibited the replication of VSV-SARS-CoV and VSV-SARS-CoV-2 viruses, as detected by measurement of eGFP expression using flow cytometry (Fig. 1*C*). CH25H also decreased wild-type VSV replication (Fig. 1*C*), as previously reported (28). We interpret the partial reduction of eGFP fluorescence intensity in the infected cells as an indication that CH25H treatment slows down the kinetics of infection. In contrast, rotavirus and adenovirus replication were not affected (Fig. 1*D*). Un-like IFIH1, CH25H expression or 25HC treatment did not induce type I or type III IFN expression (*SI Appendix*, Fig. S1*G*). The replication of a clinical isolate of SARS-CoV-2 (2019-nCoV/USA-WA1/2020 strain) also was suppressed in HEK293-hACE2 cells expressing CH25H compared to control plasmid transfection (Fig. 1*E*).

To examine the physiological relevance of CH25H antiviral activity, we used CRISPR/Cas9 to edit the endogenous *CH25H* locus. CH25H knockout was verified by both Sanger sequencing and Western blot (*SI Appendix*, Fig. S1 *H* and *I*). Genetic depletion of CH25H expression resulted in a significant increase of VSV-SARS-CoV-2 replication (Fig. 1*F*). Taking these data together, our genetic screen revealed CH25H as a host restriction factor of SARS-CoV-2 replication.

**Fig. 2.**
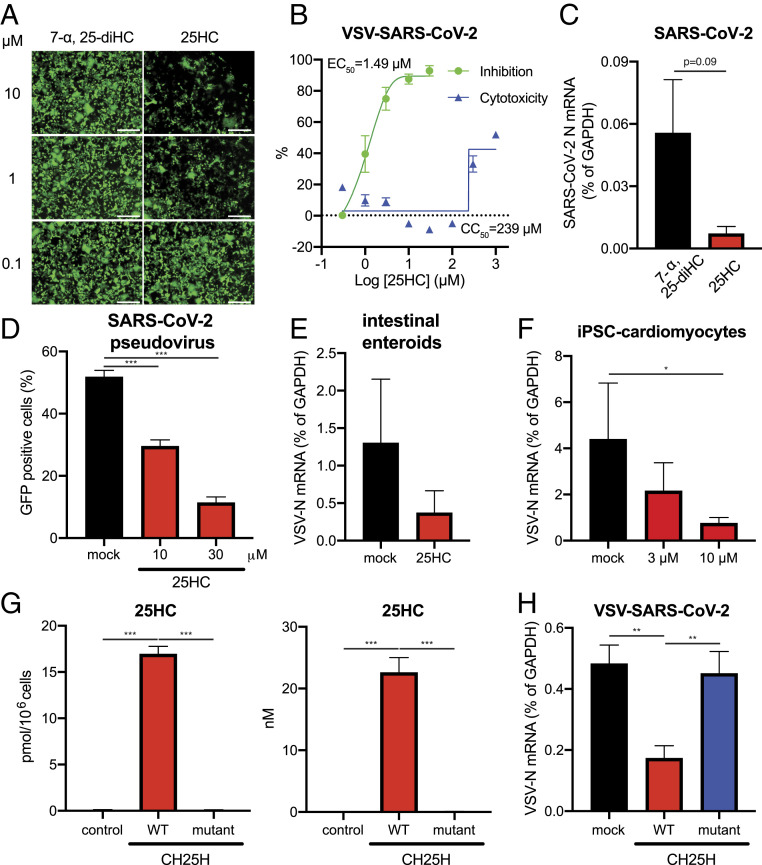
25HC inhibits SARS-CoV-2 replication. (*A*) HEK293-hACE2 cells were treated with 7-α, 25-diHC or 25HC at 0.1, 1, or 10 µM for 1 h and infected with VSV-SARS-CoV-2 (MOI = 5). GFP signals were detected at 24 hpi. (Scale bars, 200 µm.) (*B*) MA104 cells were treated with 25HC at 0.3 to 30 µM for 1 h and infected with VSV-SARS-CoV-2 (MOI = 0.1) for 24 h. GFP signals were captured by Typhoon, quantified by ImageJ, and plotted as percentage of inhibition. For CC_50_ measurement, cells were treated with inhibitors at 0.3 μM to 1 mM for 25 h and analyzed by a Cell Counting Kit 8 and BioTek ELx800 Microplate Reader. (*C*) HEK293-hACE2 cells were treated with 7-α, 25-diHC or 25HC at 10 µM for 1 h and infected with SARS-CoV-2 (MOI = 0.5). At 24 hpi, the mRNA level of SARS-CoV-2 N was measured by qRT-PCR and normalized to GAPDH expression. (*D*) HEK293T cells were transfected with ACE2 and TMPRSS2, treated with 25HC at 10 or 30 µM for 1 h, and infected with SARS-CoV-2-S-lenti-GFP pseudoviruses (1.45 × 10^4^ pg of HIV p24). GFP^+^ cells were quantified at 24 hpi. (*E*) Differentiated human duodenum enteroids in monolayer were treated with 25HC at 10 µM for 1 h and apically infected with VSV-SARS-CoV-2 (MOI = 1). At 24 hpi, the mRNA level of VSV-N was measured by qRT-PCR and normalized to GAPDH expression. (*F*) Human induced pluripotent stem cell (iPSC)-derived cardiomyocytes were treated with 25HC at 3 or 10 µM for 1 h and infected with VSV-SARS-CoV-2 (MOI = 0.01). At 96 hpi, the mRNA level of VSV-N was measured by qRT-PCR and normalized to GAPDH expression. (*G*) HEK293-hACE2 cells were transfected with wild-type or enzymatic mutant CH25H for 36 h. Cell pellets and culture supernatants were harvested for 25HC mass spectrometry. (*H*) HEK293-hACE2 cells were transfected with wild-type or enzymatic mutant CH25H for 24 h and infected with VSV-SARS-CoV-2 (MOI = 1). At 18 hpi, the mRNA level of VSV-N was measured by qRT-PCR and normalized to GAPDH expression. For all figures, experiments were repeated at least three times with similar results. Data are represented as mean ± SEM. Statistical significance is from pooled data of the multiple independent experiments (*P ≤ 0.05; **P ≤ 0.01; ***P ≤ 0.001).

Next, we tested whether the antiviral activity of CH25H depends on 25HC synthesis. As compared to the control 7-α, 25-diHC, pretreatment of HEK293-hACE2 cells with 25HC for 1 h prior to VSV-SARS-CoV-2 infection recapitulated the suppressive effect of CH25H overexpression and reduced virus replication (Fig. 2*A*). 25HC dose-dependently inhibited VSV-SARS-CoV-2 infection in MA104 cells, with an approximate EC_50_ of 1.49 µM (with 95% confidence interval of 1.09 to 2.08 µM) (Fig. 2*B*). The 50% cytotoxic concentration (CC_50_) was calculated to be at 239 µM. Thus, 25HC had an excellent selectivity index of 160. When plaque assays were performed in the presence of 25HC, there was a reduction in both plaque numbers and sizes (*SI Appendix*, Fig. S2 *A* and *B*). Similarly, wild-type SARS-CoV-2 virus replication was inhibited by 25HC treatment (Fig. 2*C*). The infectivity of a lentivirus backbone-based SARS-CoV-2 pseudovirus also was suppressed by 25HC, suggesting that the antiviral effect is restricted to early-stage virus replication (entry, transcription, translation, and so forth). We and others previously showed that primary human intestinal epithelial cells and cardiomyocytes are extrapulmonary SARS-CoV-2 target cells (7, 18, 29). Importantly, 25HC inhibited VSV-SARS-CoV-2 replication in these primary cell culture systems in a dose-dependent manner (Fig. 2 *E* and *F* and *SI Appendix*, Fig. S2*C*).

To investigate a potential 25HC-independent antiviral function of CH25H (30), we generated a CH25H catalytic mutant (H422Q and H423Q). Using liquid chromatography-mass spectrometry (LC-MS), we quantified the secreted and intracellular levels of 25HC and downstream product 7-α, 25-diHC. At similar protein levels (*SI Appendix*, Fig. S2*D*), mutant CH25H did not produce 25HC and 7-α, 25-diHC (Fig. 2*G* and *SI Appendix*, Fig. S2*E*). As expected, mutant CH25H failed to inhibit VSV-SARS-CoV-2 infection like the wild-type protein (Fig. 2*H*). Collectively, our results suggest the natural product 25HC mediates the antiviral activity of CH25H and restricts SARS-CoV-2 virus infection.

**Fig. 3.**
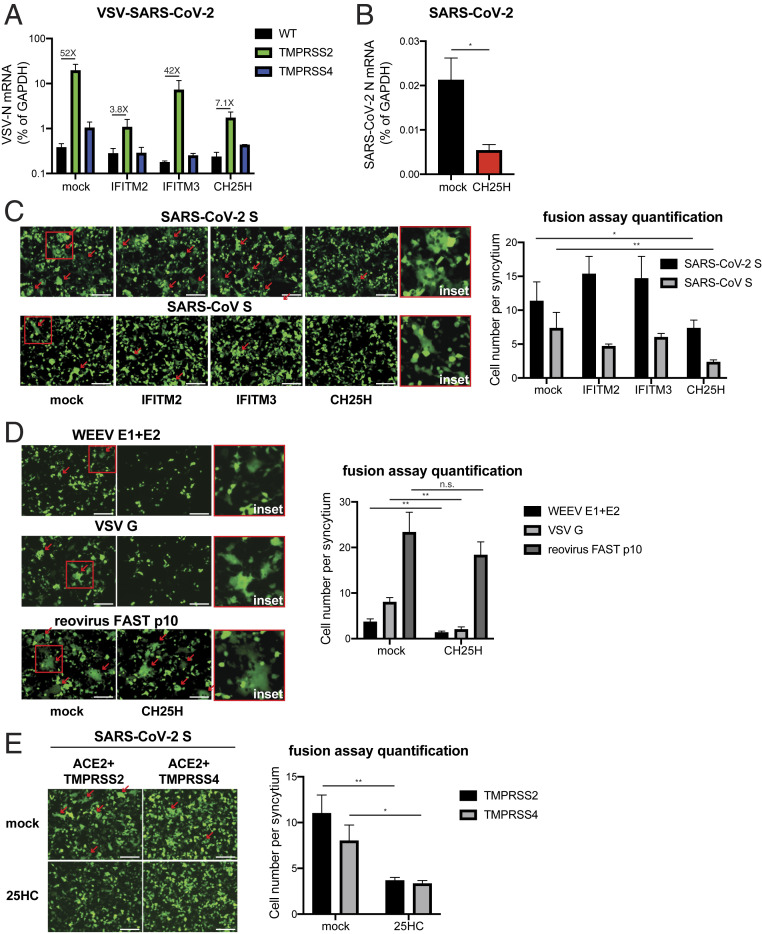
CH25H and 25HC block SARS-CoV-2 S-mediated membrane fusion. (*A*) Wild-type HEK293-hACE2 cells with or without TMPRSS2 or TMPRSS4 expression were transfected with mock, IFITM2, IFITM3, or CH25H for 24 h and infected with VSV-SARS-CoV-2 (MOI = 1). At 24 hpi, the mRNA level of VSV N was measured by qRT-PCR and normalized to GAPDH expression. (*B*) HEK293-hACE2-TMPRSS2 cells with or without CH25H expression were infected with a clinical isolate of SARS-CoV-2 (MOI = 0.5). At 24 hpi, the mRNA level of SARS-CoV-2 N was measured by qRT-PCR and normalized to GAPDH expression. (*C*) HEK293-hACE2-TMPRSS2 cells were cotransfected with eGFP, either SARS-CoV S or SARS-CoV-2 S, and IFITM2, IFITM3, or CH25H for 24 h. The red arrows highlight the syncytia formation. Enlarged images of mock condition are highlighted by red boxes and included as *Insets* (magnification, 160-fold). (Scale bars, 200 µm.) The number of cells in each GFP^+^ syncytia was quantified based on the six brightest syncytia per image. (*D*) HEK293 cells were cotransfected with eGFP, Western equine encephalomyelitis virus (WEEV) E1 and E2, VSV G, or reovirus FAST p10, with or without CH25H for 24 h. The red arrows highlight the syncytia formation. Enlarged images of mock condition are highlighted by red boxes and included as *Insets* (magnification, 160-fold). (Scale bars, 200 µm.) Quantification of membrane fusion assays was performed by calculating the number of cells in GFP^+^ syncytia. (*E*) HEK293-hACE2 cells stably expressing TMPRSS2 or TMPRSS4 were cotransfected with SARS-CoV-2 S and eGFP with or without 25HC (10 µM) for 24 h. The red arrows highlight the syncytia formation. (Scale bars, 200 µm.) Quantification of membrane fusion assays was performed by calculating the number of cells in GFP^+^ syncytia. For all figures, experiments were repeated at least three times with similar results. Data are represented as mean ± SEM. Statistical significance is from pooled data of the multiple independent experiments (*P ≤ 0.05; **P ≤ 0.01; n.s., not significant).

During SARS-CoV-2 entry into host cells, S protein binding to ACE2 enables its cleavage by membrane-bound TMPRSS serine proteases and subsequent fusion of the viral membrane to the host cell membrane (15, 18, 31). Previous work suggests that trypsin treatment or TMPRSS2 expression alleviates IFITM-mediated restriction of SARS-CoV and HCoV-229E entry (32, 33). Furthermore, TMPRSS2 is abundantly expressed in human nasal and intestinal epithelial cells (18, 34). Thus, we examined whether the presence of TMPRSS2 assists VSV-SARS-CoV-2 to overcome ISG restriction. TMPRSS2 expression enhanced VSV-SARS-CoV and VSV-SARS-CoV-2 infection at 6 hpi (*SI Appendix*, Fig. S3*A*), compared to control HEK293-hACE2 cells (Fig. 1*C*). Un-like IFITM3, CH25H partially retained its antiviral activity and led to reduced VSV-SARS-CoV-2 replication in TMPRSS2-expressing cells (Fig. 3*A*). Similarly, wild-type SARS-CoV-2 replication was inhibited by CH25H and 25HC in TMPRSS2-expressing cells (Fig. 3*B*).

We next examined the effect of 25HC on SARS-CoV S and SARS-CoV-2 S mediated membrane fusion, since 25HC blocks cell fusion by Nipah F and VSV G proteins (28), which are class I and class III viral fusion proteins, respectively (35). We set up an in vitro cell-to-cell fusion assay based on the expression of S, eGFP, ACE2, and TMPRSS2 in HEK293 cells, independent of virus infection (Fig. 3*C*). CH25H expression substantially reduced syncytia formation mediated by SARS-CoV-2 S (Fig. 3*C*). Although IFITM2 and IFITM3 inhibited VSV-SARS-CoV-2 replication (Fig. 1 *A* and *B*), neither prevented S-mediated fusion (Fig. 3*C*), suggesting a distinct mode of antiviral action. Compared to SARS-CoV-2 S, SARS-CoV S induced weaker cell fusion, as recently reported (36), and this process was also blocked by CH25H expression (Fig. 3*C*). CH25H also inhibited the syncytia formation induced by Western equine encephalitis virus glycoproteins (class II) and VSV-G (class III) but not reovirus FAST p10 (class IV) fusion protein (Fig. 3*D*). To mimic the virus–cell membrane fusion, we cotransfected SARS-CoV-2 S and GFP into donor cells and mixed at 1:1 ratio with ACE2^+^TMPRSS2^+^TdTomato cotransfected target cells. As expected, we observed robust syncytia formation under mock conditions (*SI Appendix*, Fig. S3*B*). CH25H expression in “recipient” cells almost completely abolished cell–cell fusion (*SI Appendix*, Fig. S3*B*). Exogenous 25HC treatment phenocopied CH25H expression and blocked SARS-CoV-2 S-mediated syncytia formation (Fig. 3*E* and *SI Appendix*, Fig. S3*B*). Similar to CH25H, 25HC failed to inhibit reovirus FAST p10-mediated fusion (*SI Appendix*, Fig. S3*C*).

To define the underlying antiviral mechanisms of the IFN-CH25H-25HC axis further, we investigated whether 25HC acts on viral or host membranes. Preincubation of VSV-SARS-CoV-2 with 10 µM of 25HC for 20 min had no effect on infectivity, as opposed to the pretreatment of host cells (*SI Appendix*, Fig. S4*A*). The timing of 25HC addition suggests it primarily acted at the entry stage of VSV-SARS-CoV-2 replication (*SI Appendix*, Fig. S4*B*). We examined a series of early events and excluded possible effects of 25HC on: 1) ACE2 surface levels; 2) S cleavage by TMPRSS2; 3) lipid raft localization, stained by a fluorophore-conjugated cholera toxin subunit B; 4) plasma membrane fluidity, stained by 6-dodecanoyl-2-dimethylamino naphthalene (Laurdan) (37); 5) endosomal pH; and 6) its ability to directly bind to recombinant SARS-CoV-2 S protein (*SI Appendix*, Fig. S4 *C* and *D*).

**Fig. 4.**
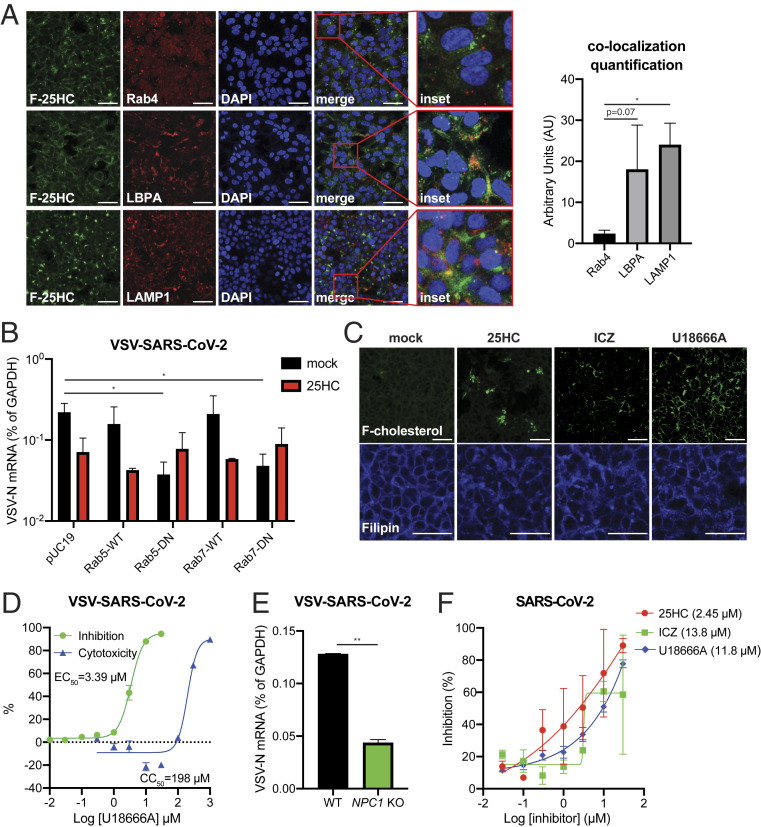
25HC inhibits endosomal cholesterol export to block SARS-CoV-2 fusion. (*A*) HEK293 cells were incubated with C4 TopFluor-25HC (F-25HC, 2 µM) for 1 h, fixed, and stained for recycling endosome (Rab4), late endosome (LBPA), lysosome (LAMP1), and nucleus (blue, DAPI) (magnification, 800-fold). (Scale bars, 30 µm.) Quantification of colocalization was calculated by Volocity. (*B*) HEK293-hACE2 cells were transfected with wild-type (WT) or dominant-negative (DN) mutants of Rab5 or Rab7 for 24 h and infected with VSV-SARS-CoV-2 (MOI = 1) with or without 25HC (10 µM). At 24 hpi, the mRNA level of VSV N was measured by qRT-PCR and normalized to GAPDH expression. (*C*) HEK293 cells were treated with TopFluor-cholesterol (F-cholesterol, 2 µM) for 1 h (*Upper*) or stained with 50 µg/mL filipin for 30 min (*Lower*) with or without 25HC (10 µM), ICZ (5 µM), or U18666A (5 µM). (Scale bars, 30 µm.) (*D*) MA104 cells were treated with U18666A at 0.01 to 30 µM for 1 h and infected with VSV-SARS-CoV-2 (MOI = 0.1) for 24 h. GFP signals were captured by Typhoon, quantified by ImageJ, and plotted as percentage of inhibition. (*E*) HEK293-hACE2-TMPRSS2 cells with or without NPC1 expression were infected with VSV-SARS-CoV-2 (MOI = 1). At 24 hpi, the mRNA level of VSV N was measured by qRT-PCR and normalized to GAPDH expression. (*F*) Vero E6 cells were treated with 25HC, ICZ, or U18666A at indicated concentrations for 1 h and infected with recombinant SARS-CoV-2-mNeonGreen virus (MOI = 0.5) for 24 h. Cells were fixed and green signals were scanned with Typhoon and quantified by ImageJ. EC_50_ values for each compound are provided in the parentheses. For all figures, experiments were repeated at least three times with similar results. Data are represented as mean ± SEM. Statistical significance is from pooled data of the multiple independent experiments (*P ≤ 0.05; **P ≤ 0.01).

Both 23-(Dipyrrometheneboron difluoride)-24-norcholesterol (TopFluor-cholesterol) and [4-(dipyrrometheneboron difluoride) butanoyl]-25-hydroxycholesterol (C4 TopFluor-25HC) are chemically fluorescently labeled cholesterol and 25HC derivatives that have been used to study membrane incorporation and lipid metabolism (38). C4 TopFluor-25HC retained an almost identical anti–VSV-SARS-CoV-2 activity as unmodified 25HC (*SI Appendix*, Fig. S4*E*) and blocked SARS-CoV-2 S-induced syncytia formation (*SI Appendix*, Fig. S4*F*), enabling us to use it as a tool to probe the antiviral mechanism of 25HC. Upon host cell uptake, C4 TopFluor-25HC exhibited punctate patterns and partially colocalized with lysobisphosphatidic acid (LBPA)-positive late endosomes and LAMP1^+^ lysosomes but not Rab4^+^ recycling endosomes (Fig. 4*A*). Thus, we hypothesized that SARS-CoV-2 depends on endosomal trafficking to establish active replication. Consistent with this hypothesis, ectopic expression of Rab5 and Rab7 dominant-negative mutants but not the wild-type proteins significantly decreased VSV-SARS-CoV-2 infection (Fig. 4*B* and *SI Appendix*, Fig. S4*G*). However, Rab5 and Rab7 mutants did not have an additive effect with 25HC treatment (Fig. 4*B*), further suggesting that 25HC may act at these endosomal vesicles.

25HC is capable of binding Niemann-Pick C1 (NPC1) in vitro (39), which is responsible for the egress of cholesterol from the endosomal/lysosomal compartment (40). 25HC treatment led to an accumulation of intracellular TopFluor-cholesterol (Fig. 4*C*). We also observed that in cells stained with filipin, which labels unesterified cholesterol and preferentially recognized cholesterol over 25HC (*SI Appendix*, Fig. S5*A*), a cholesterol accumulation by 25HC and NPC1 inhibitors itraconazole (ICZ) and U18666A (Fig. 4*C*). ICZ treatment significantly reduced VSV-SARS-CoV-2 titers (*SI Appendix*, Fig. S5*B*). The antiviral activity of 25HC and ICZ depended on cholesterol accumulation in the endosomal/lysosomal compartment because the inhibition was markedly diminished in serum-free media that is devoid of cholesterol (*SI Appendix*, Fig. S5*C*). Consistent with our model, pharmacological inhibition of NPC1 by U18666A or genetic depletion of *NPC1* by CRISPR/Cas9 potently restricted VSV-SARS-CoV-2 replication (Fig. 4 *D* and *E* and *SI Appendix*, Fig. S5*D*). In contrast to chloroquine and camostat, both of which are antiviral but through different mechanisms, 25HC, ICZ, or U18666A efficiently reduced SARS-CoV-2 S-mediated cell–cell fusion (*SI Appendix*, Fig. S5*E*). Finally, all three compounds suppressed the replication of a recombinant SARS-CoV-2 virus that encodes an mNeon-Green reporter (41) in Vero E6 cells (Fig. 4*F*). Collectively, our data support a model that 25HC suppresses SARS-CoV-2 S protein-mediated fusion, which inhibits virus replication, possibly by altering cholesterol levels.

The identification of ISGs against different virus families have provided invaluable insights into both virus entry pathways and host innate immune system evolution (42–45). To date, few ISGs have been identified that restrict SARS-CoV replication [IFITMs (33) and GILT (46)] and SARS-CoV-2 replication [LY6E (20, 21), IFITMs (21, 22), and ZAP (47)]. Here, we present evidence that IFN-inducible *CH25H* and its natural product 25HC restrict S-mediated membrane fusion and block SARS-CoV-2 entry into host cells. 25HC has shown broad antiviral activity against a wide range of enveloped viruses (26, 28, 30, 48) and nonenveloped viruses, such as reovirus (49) and murine norovirus (50). 25HC treatment did not inhibit the infectivity of rhesus rotavirus RRV strain (Fig. 1*D*), in contrast to the inhibition previously seen with the human rotavirus Wa strain (51), possibly because they differ in the endosomal compartment from which they exit into the cytosol (52). There seem to be two modes of inhibitory mechanisms involved. One requires a high micromolar concentration and more than 6 h of preincubation time to be effective, in the case of reovirus (49), pseudorabies virus (53), and human papillomavirus-16 (54), suggesting an indirect metabolic/cellular pathway-mediated mechanism, whereas the other—which includes influenza A virus (26), Lassa fever virus (55), hepatitis C virus (56), and SARS-CoV-2 (Fig. 2)—functions at a low-micromolar/high-nanomolar range.

Combined with the recent report that apilimod, a PIKfyve kinase inhibitor, effectively inhibits SARS-CoV-2 infection (57), we confirm that this virus reaches late endosomal compartment for membrane fusion and access to the cytosol, at least in ACE2^+^TMPRSS2^−^ cells. Recently, two independent studies also reported the anti–SARS-CoV-2 activity of 25HC (58, 59). In the second study, Wang et al. (59) observed depletion of cholesterol at the plasma membrane by 25HC treatment, which is consistent with our findings of SARS-CoV-2 S protein-mediated fusion blockade (Fig. 3 *C* and *E*). Taken together, our data suggest a unifying model in which 25HC results in a redistribution of cholesterol and inhibits both endosomal entry and plasma membrane fusion, which potentially explains the CH25H inhibitory activity against wild-type SARS-CoV-2 in both TMPRSS2^−^ and TMPRSS2^+^ cells (Fig. 1 *C* and *E* and *SI Appendix*, Fig. S3 *A* and *B*).

One point worth noting is that wild-type VSV is sensitive to 25HC antiviral effect (Fig. 1*C*), while its entry is not dependent on NPC1 (60). Therefore, in the case of SARS-CoV-2, it is plausible that the reduced virus infection in the absence of NPC1 is uncoupled from 25HC antiviral mechanism of action. The latter is likely multifactorial and involves other cholesterol-binding proteins.

Another question that warrants further investigation is whether 25HC interferes with other steps in the SARS-CoV-2 replication cycle. While we observed a relatively weak inhibition of infection at early time points, such as 6 hpi (Fig. 1*C* and *SI Appendix*, Figs. S3*A* and S4*A*), the antiviral effect is magnified by 24 hpi (Figs. 1 *D* and *E*, 2*C*, and 4*F*). It will be of interest to examine whether this reflects an antagonism of viral particle maturation or the release or infectivity of virus progeny.

Our data instruct potential drug combinations of 25HC in conjunction with those targeting the cytoplasmic steps of the SARS-CoV-2 replication cycle, such as its main protease (61, 62) or polymerase (63). Further in vivo studies in animal models of SARS-CoV-2 infection and pathogenesis are required to establish the physiological impact of 25HC-based drugs or compounds that modulate antiviral activities.

## Materials and Methods

### Plasmids, Cells, Reagents, and Viruses.

#### Plasmids.

*CH25H*, *IFIH1*, *IFITM1*, *IFITM2*, *IFITM3*, and *LY6E* were cloned into pLX304 lentiviral vector with a C-terminal V5 tag. Human ACE2 was cloned into pDEST-mCherry vector with an N-terminal mCherry tag. TMPRSS2 and TMPRSS4 plasmids were used as previously described (18). Single-guide RNA against *CH25H* or *NPC1* (*SI Appendix*, Table S1) was cloned into lenti-CRISPR_v2 vector (Addgene, #52961). *CH25H* point mutations were introduced by QuikChange II site-directed mutagenesis (Agilent, #200524) using primers in *SI Appendix*, Table S1. GFP-tagged Rab5 and Rab7 constructs were used as reported previously (64). Codon-optimized SARS-CoV-2 S was a kind gift from Nevan Krogan at the University of California, San Francisco, CA (65). pCAGGS-SARS-CoV S was a kind gift of Paul Bates at the University of Pennsylvania, Philadelphia, PA (66). pMIG-WEEV-IRES-GFP plasmid was generated by Zhuoming Liu in the S.P.J.W. laboratory at the Washington University School of Medicine in St. Louis, St. Louis, MO. PM-GFP and VSV-G plasmids were obtained from Addgene (#21213 and #12259, respectively). pCAGGS-FAST-p10 from pteropine orthoreovirus was generated in the Kobayashi laboratory at the Osaka University, Japan (67). pEGFP-N1 and pCMV-TdTomato were obtained from Clontech.

#### Cells.

Human embryonic kidney cell line HEK293 (CRL-1573) were originally obtained from American Type Culture Collection (ATCC) and cultured in complete DMEM. Rhesus kidney epithelial cell lines MA104 cells were cultured in complete M199 medium. HEK293T, Vero cells (CCL81, ATCC), and Vero E6 cells (CRL-1586, ATCC) were cultured in complete DMEM (Corning). HEK293-hACE2-mCherry cells were cultured in complete DMEM with G418 addition (500 μg/mL). HEK293-hACE2 cells and HEK293 cells stably expressing ACE2 and TMPRSS2 were used as previously described (18). For overexpression, HEK293-hACE2 cells were transduced with lentiviruses encoding *CH25H* for 2 d in the presence of polybrene (8 µg/mL) and cultured in DMEM containing 5 µg/mL of blasticidin. For CRISPR knockout, HEK293-hACE2-TMPRSS2 cells were transduced with lentiviruses encoding Cas9 and single-guide RNA against *CH25H* or *NPC1* for 2 d and cultured in the presence of puromycin (1 µg/mL).

#### Reagents.

25HC, 7-α, 25-diHC, trypsin, U18666A, and filipin were purchased from Sigma-Aldrich. C4 TopFluor- 25HC and TopFluor-cholesterol were purchased from Avanti Polar Lipids. The 6-Dodecanoyl-2-dimethylaminonaphthalene (Laurdan, D250), cholera toxin subunit B (C34777), and pHrodo AM Variety Pack (P35380) were purchased from Thermo Fisher. ICZ and camostat were purchased from Selleck Chemicals. Chloroquine (tlrl-chq) was purchased from Invivogen. The viability of MA104 cells after drug treatment was determined using the Cell Counting Kit 8 (ab228554, Abcam).

#### Viruses.

Recombinant VSV-eGFP-SARS-CoV-2 was previously described (14). VSV-eGFP-SARS-CoV was constructed in a similar manner. Adenovirus (serotype 5) and rotavirus (rhesus RRV strain) were propagated and used as previously described (68). A clinical isolate of SARS-CoV-2 (2019-nCoV/USA-WA1/2020 strain) was obtained from Natalie Thornburg at the Centers for Disease Control and Prevention, Atlanta, GA. An mNeonGreen SARS-CoV-2 reporter virus was used as previously reported (41). SARS-CoV-2 viruses were passaged in Vero CCL81 cells and titrated by focus-forming assay on Vero E6 cells. Plaque assays were performed in MA104 cells seeded in six-well plates using an adapted version of the rotavirus plaque assay protocol (69). The plaque plates were scanned by Amersham Typhoon 5 (GE) and diameters were measured by ImageJ (NIH).

### Identification and Quantification of Oxysterols.

Oxysterols were extracted from pellets (3 × 10^6^ cells) by adding 1 mL of absolute ethanol containing 20 ng of [^2^H_7_]24R/S-hydroxycholesterol, 20 ng [^2^H_7_]22S-hydroxycholest-4-en-3-one (converted from [^2^H_7_]22S-hydroxycholesterol by cholesterol oxidase) and 400 ng of [^2^H_7_]cholesterol (all sterols from Avanti Polar Lipids) with sonication in an ultrasonic bath (5 min). The solution was diluted to give 1.5 mL of 70% ethanol. To extract secreted oxysterols from the culture medium, medium (1 mL) was added drop-wise, with sonication in an ultrasonic bath (5 min), to 2.3 mL of absolute ethanol containing the same amount of internal standards, as above, to give a solution of 3.3 mL of 70% ethanol. The oxysterols were then separated from cholesterol, derivatized with Girard P reagent and analyzed by LC-MS(MS^n^) on a Orbitrap Elite, as described previously (70). Identification was performed by comparing retention time, exact mass, and MS^n^ spectra to authentic standards and quantification was performed by stable isotope dilution using reconstructed ion-chromatograms generated in the Orbitrap.

### Cardiomyocyte Infection.

Human induced pluripotent stem cell-derived cardiomyocytes (R1117) were purchased from Cellular Dynamics International and seeded onto a 24-well plate coated with 0.1% gelatin (07903, Stemcell Technologies) at 1.36 × 10^5^ cells per well, following manufacturer’s recommendations. The cells were seeded in culture (37 °C, 5% CO_2_) for 48 h in plating medium and then changed to maintenance media, which was replaced every 2 d. VSV-SARS-CoV-2 infections were performed at day 6 after cell seeding.

### SARS-CoV-2-Lenti-GFP Pseudovirus Preparation.

Confluent HEK293T cells in six-well plates were cotransfected with plasmids pLenti-CMV-GFP-Puro (Addgene #17448) (71), psPAX2 (Addgene #12260), and pcDNA3.1-SARS-CoV-2-spike (Addgene #145032) (72) with a mass ratio of 4:3:1 (1.6 μg, 1.2 μg, and 0.4 μg, respectively) by Lipofectamine 3000 (Life Technologies). The supernatant was collected at days 2, 3, and 4 posttransfection and combined. To concentrate the pseudovirus particles from the collected supernatant, polyethylene glycol (PEG)-8000 powder was added to a final concentration (wt/vol) of 10% and the mixture was gently rotated at 4 °C overnight, followed by centrifugation at 1,000 × *g* for 30 min at 4 °C. The pellet was dissolved in serum-free DMEM/F12 medium with 1% volume of the original supernatant samples. The concentrated virus stock was aliquoted and stored at −80 °C. For HIV-1 p24 titration assay, the concentrated pseudovirus particles were diluted in PBS (or DMEM/F12 medium) by 10,000-fold and subjected to the titration assay by using HIV-1 p24 quantitation ELISA Kit (Abcam) according to the manufacturer's directions.

### RNA Extraction and Quantitative PCR.

Total RNA was extracted from cells using RNeasy Mini kit (Qiagen) and reverse transcription was performed with High Capacity RT kit and random hexamers, as previously described (73). Quantitative PCR was performed using the AriaMX (Agilent) with a 25-µL reaction, composed of 50 ng of cDNA, 12.5 µL of Power SYBR Green master mix or Taqman master mix (Applied Biosystems), and 200 nM both forward and reverse primers. All SYBR Green primers and Taqman probes used in this study are listed in *SI Appendix*, Table S1.

### Flow Cytometry.

HEK293-hACE2 or HEK293-hACE2-TMPRSS2 cells with or without CH25H expression were inoculated with wild-type VSV-GFP, VSV-SARS-CoV, or VSV-SARS-CoV-2 at a multiplicity of infection (MOI) = 10 (based on titers in Vero cells) for 1 h at 37 °C. At 6 hpi, cells were harvested and fixed in 4% PFA. GFP^+^ cells were determined by BD LSRFortessa X-20 cell analyzer and analyzed by FlowJo v10.6.2 (BD).

### Brightfield and Immunofluorescence Microscopy.

For brightfield and epifluorescence, cultured cells were imaged by REVOLVE4 microscope (ECHO) with a 10× objective. For confocal microscopy, samples in eight-well chamber slides were fixed in 4% paraformaldehyde for 10 min at room temperature and stained as previously described (74). Cells were permeabilized and stained with antibodies against DAPI (P36962, Thermo Fisher), LAMP1 (9091S, Cell Signaling), LBPA (MABT837, Sigma), and Rab4 (ab13252, Abcam) at room temperature for 1 h. For filipin staining, cells were fixed and incubated with 50 μg/mL filipin (SAE0087, Sigma-Aldrich) at 37 °C for 30 min. Stained cells were washed with PBS, whole-mounted with Antifade Mountant, and imaged with a Zeiss LSM880 Confocal Microscope at the Molecular Microbiology imaging core facility at Washington University in St. Louis. Images were visualized by Volocity v6.3 and quantification was determined by ImageJ (NIH).

### Western Blotting.

Cell lysates were harvest in RIPA buffer supplemented with protease inhibitor mixture and phosphatase inhibitor. Proteins were resolved in SDS/PAGE and analyzed by antibody as described previously (75) using the following antibodies and dilutions: ACE2 (MAB933, R&D Systems), GAPDH (631402, BioLegend), GFP (2555S, Cell Signaling), NPC1 (ab124801, Abcam), SARS-CoV-2 S1 (40590-T62, Sino Biological), SARS-CoV-2 S2 (40592-T62, Sino Biological), and V5 (13202S, Cell Signaling). Secondary antibodies were anti-rabbit (7074, Cell Signaling) or anti-mouse (7076, Cell Signaling) IgG horseradish peroxidase-linked antibodies. Protein bands were visualized with Clarity ECL substrate and a Bio-Rad Gel Doc XR system.

### Statistical Analysis.

All bar graphs were displayed as means ± SEM. Statistical significance in the data in Figs. 1 *E* and *F*, 2*C*, 3*B*, and 4*E* and *SI Appendix*, Figs. S1 *C* and *D*, S2*B*, and S5*B* was calculated by Student’s *t* test using Prism 8.4.2 (GraphPad). Statistical significance in the data in Figs. 1 *B* and *D*, 2 *D*, *F*–*H*, 3 *C*–*E*, and 4 *A* and *B* and *SI Appendix*, Figs. S1*G*, S2*E*, S3 *B* and *C*, S4*A*, and S5*E* was calculated by pairwise ANOVA using Prism 8. Nonlinear regression (curve fit) was performed to calculate EC_50_ and CC_50_ values for Figs. 2*B* and 4 *D* and *F* and *SI Appendix*, Fig. S4*E*. All data are presented as *P ≤ 0.05, **P ≤ 0.01, and ***P ≤ 0.001. All experiments other than Fig. 1*A* were repeated at least three times. Fig. 1*A* was performed twice with average numbers indicated on the graph. The raw data are included in Dataset S1.

## Supplementary Material

Supplementary File

Supplementary File

## Data Availability

All study data are included in the article and supporting information.
