# Supplementary material for: Cholesterol 25-hydroxylase suppresses SARS-CoV-2 replication by blocking membrane fusion
Source: Proc Natl Acad Sci U S A. 2020 Nov 25;117(50):32105–13. doi: 10.1073/pnas.2012197117 (PMC7749331; doi:10.1073/pnas.2012197117)
Supplement: Supplementary File [file pnas.2012197117.sapp.pdf]

Supplementary Information for

Cholesterol 25-hydroxylase suppresses SARS-CoV-2 replication by blocking membrane fusion

Ruochen Zang, James Brett Case, Eylan Yutuc, Xiucui Ma, Sheng Shen, Maria Florencia Gomez Castro, Zhuoming Liu, Qiru Zeng, Haiyan Zhao, Juhee Son, Paul W. Rothlauf, Alex J.B. Kreutzberger, Gaopeng Hou, Hu Zhang, Sayantan Bose, Xin Wang, Michael D. Vahey, Kartik Mani, William J. Griffiths, Tom Kirchhausen, Daved H. Fremont, Haitao Guo, Abhinav Diwan, Yuqin Wang, Michael S. Diamond, Sean P.J. Whelan, Siyuan Ding

This PDF file includes:

SI Figs. 1 to 5 with figure legends

SI Table S1

Supplemental Figures 1-5 and Figure Legends

Figure S1

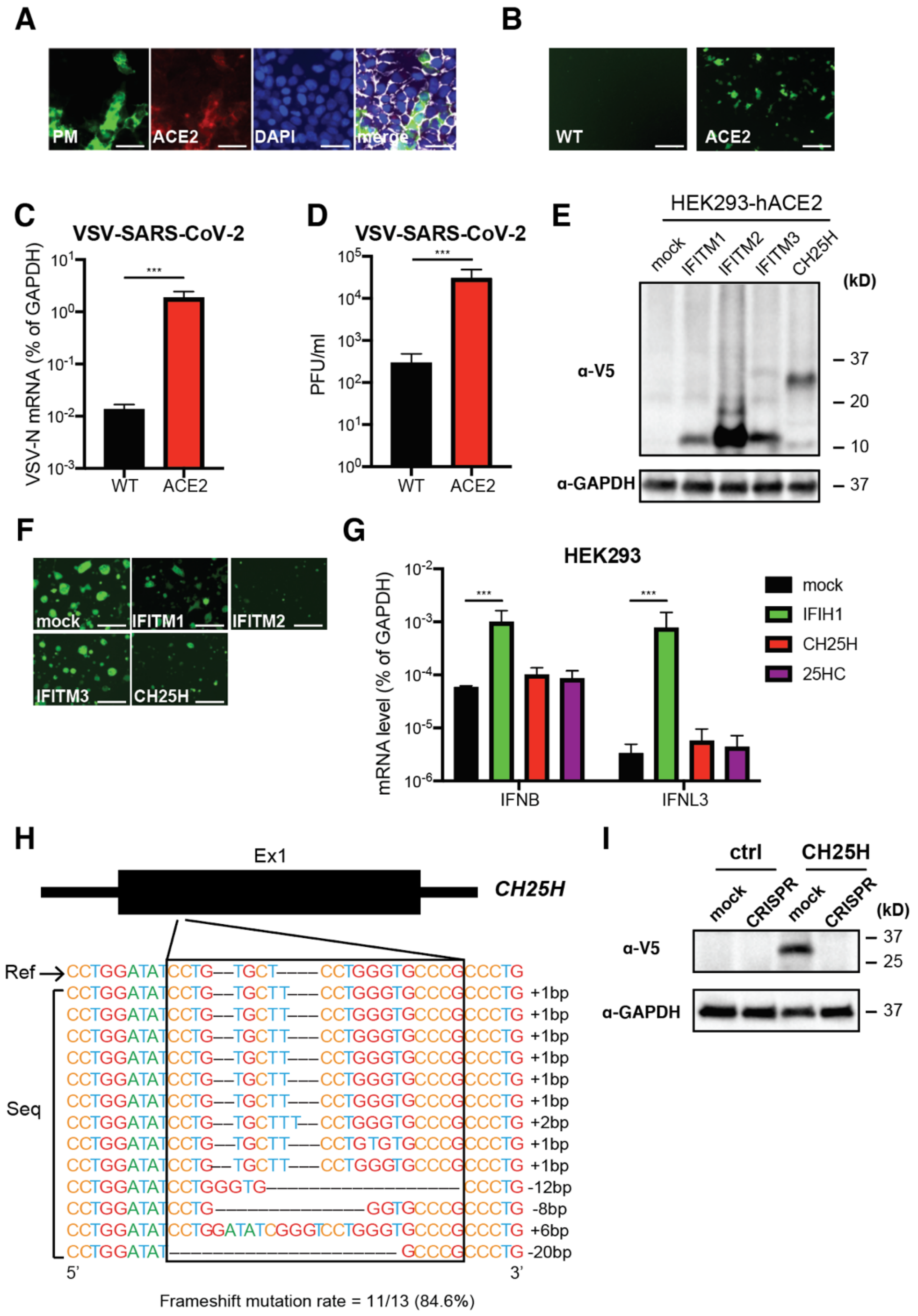

Fig. S1. CH25H suppresses VSV-SARS-CoV-2 replication in HEK293-hACE2 cells.

(A) HEK293-hACE2-mCherry cells were transfected with plasma membrane (PM)-localized GFP and stained for cell surface (green), ACE2 (red), nucleus (DAPI, blue), and actin (white). Scale bar: 30 μm.

(B) Wild-type (WT) HEK293 or HEK293-hACE2-mCherry cells were infected with VSV-SARS-CoV-2 (MOI=1) for 8 hr. Scale bar: 200 μm.

(C) Same as (B) except that infection was 24 hr and RNA was harvested for RT-qPCR measuring the mRNA level of VSV N compared to GAPDH expression.

(D) Same as (B) except that infection was 24 hr and cell lysates were harvested for plaque assays.

(E) HEK293-hACE2 cells stably expressing indicated ISGs were harvested for western blot and probed for V5-tagged ISG and GAPDH protein levels.

(F) HEK293-hACE2 cells stably expressing indicated ISGs were infected with VSV-SARS-CoV-2 (MOI=1) for 24 hr. Scale bar: 200 μm.

(G) HEK293 cells were transfected with mock, IFIH1, or CH25H plasmids for 24 hr or treated with 25HC (10 μM) for 1 hr. RNA was harvested and the mRNA levels of IFN-β (IFNB) and IFN-λ (IFNL3) were measured by RT-qPCR and normalized to GAPDH expression.

(H) HEK293-hACE2-CH25H cells transduced with lentiviruses encoding Cas9 and single-guide RNA against *CH25H* were harvested for Sanger sequencing to examine the edited *CH25H* locus.

(I) Same as (H) except that western blot was performed instead to examine the protein levels of CH25H and GAPDH.

For all figures, experiments were repeated at least three times with similar results. Data are represented as mean ± SEM. Statistical significance is from pooled data of the multiple independent experiments (***$p \leq 0.001$).

Figure S2

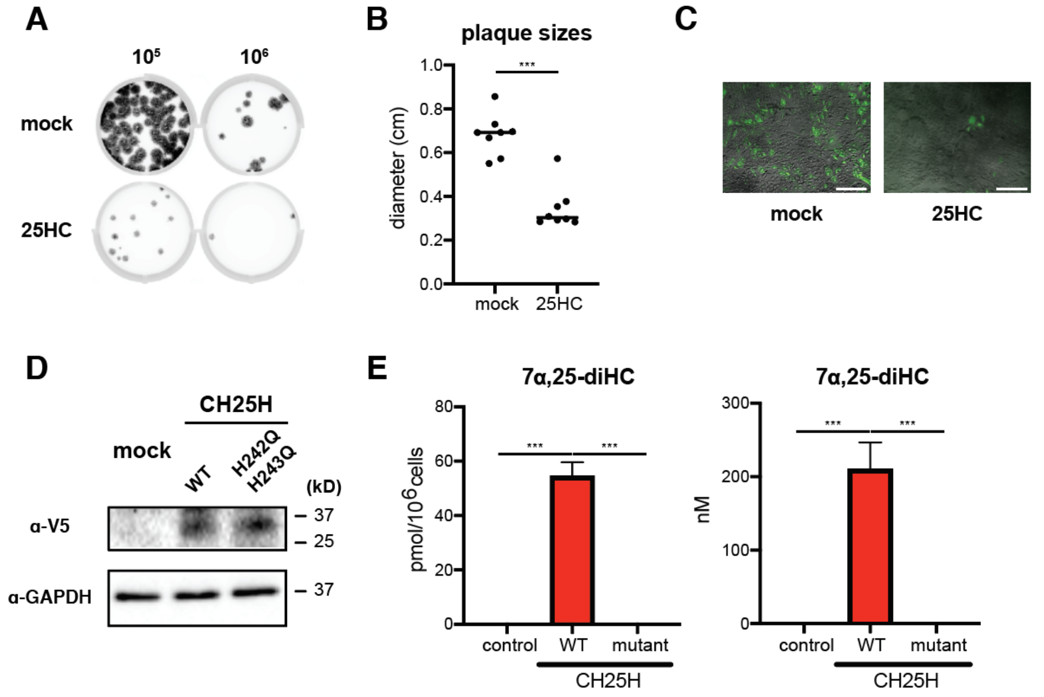

Fig. S2. 25HC restricts VSV-SARS-CoV-2 replication in MA104 cells.

(A) MA104 cells were infected with serially diluted VSV-SARS-CoV-2 with or without 25HC (10 μM). At 3 dpi, GFP signals were scanned with Typhoon.

(B) Quantification of plaque sizes in (A).

(C) Human iPSC-derived cardiomyocytes were treated with 25HC (10 μM) for 1 hr and infected with VSV-SARS-CoV-2 (MOI=0.01). GFP signals were detected at 96 hpi. Scale bar: 200 μm.

(D) HEK293-hACE2 cells were transfected with wild-type (WT) or enzymatic mutant CH25H for 24 hr. Cell lysates were harvested for western blot examining CH25H and GAPDH protein levels.

(E) HEK293-hACE2 cells were transfected with WT or enzymatic mutant CH25H for 36 hr. Cell pellets and culture supernatants were harvested for 7-α, 25-diHC mass spectrometry.

For all figures, experiments were repeated at least three times with similar results. Statistical significance is from pooled data of the multiple independent experiments (***$p \leq 0.001$).

Figure S3

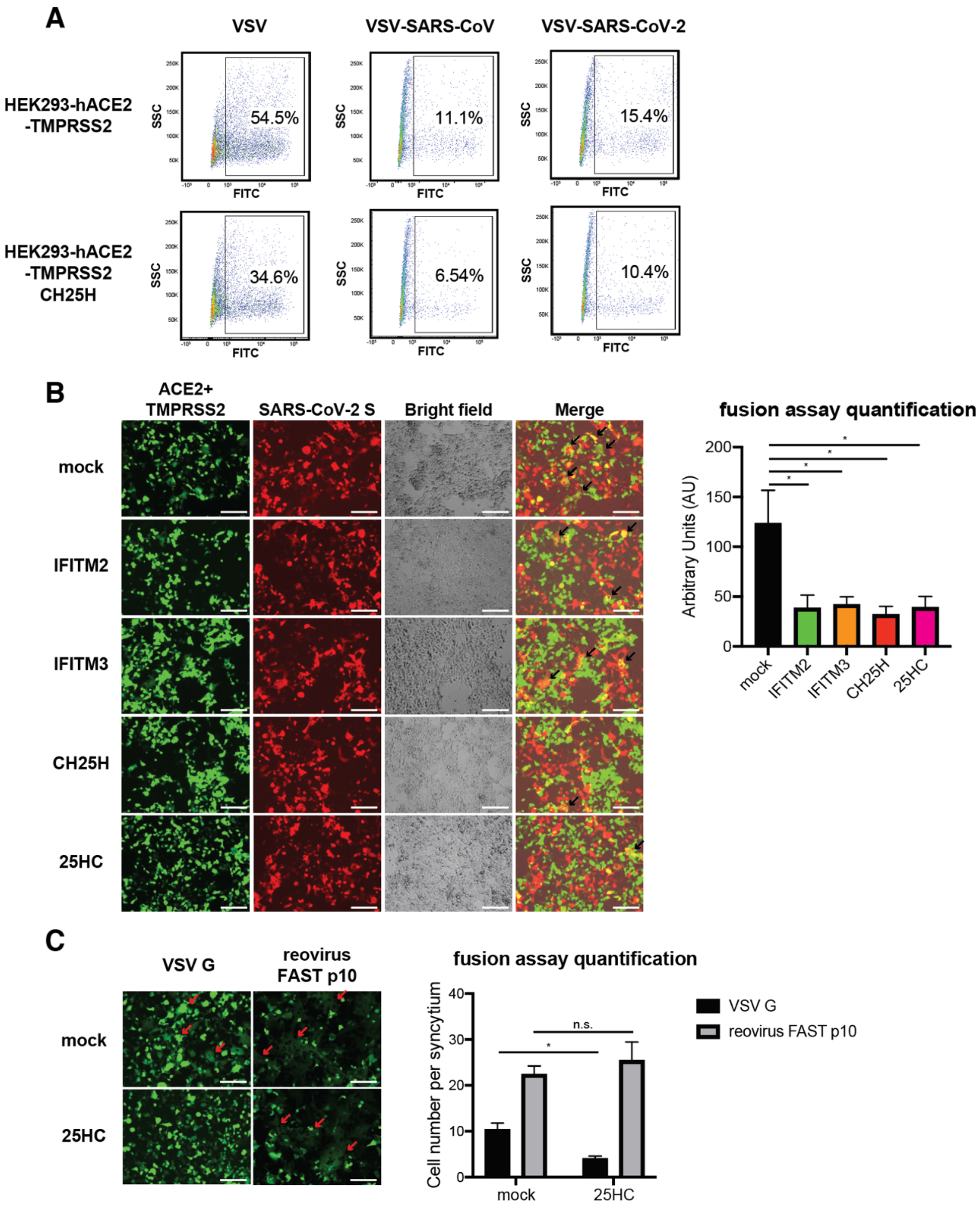

Fig. S3. CH25H and 25HC block SARS-CoV-2 S mediated fusion.

(A) HEK293-hACE2-TMPRSS2 cells were infected with wild-type VSV, VSV-SARS-CoV or VSV-SARS-CoV-2 (MOI=10) for 6 hr. Cells were harvested and measured for GFP percentage and intensity by flow cytometry.

(B) HEK293-hACE2-TMPRSS2 cells expressing eGFP and indicated ISGs or treated with 25HC (10 μM) were mixed at 1:1 ratio and co-cultured with HEK293 cells expressing SARS-CoV-2 S and TdTomato for 24 hr. Note the formation of cell-cell fusion (yellow), highlighted by black arrows. Scale bar: 200 μm. Quantification of membrane fusion assays was performed by calculating the yellow signals in the images.

(C) HEK293 cells were co-transfected with eGFP, VSV G, or reovirus FAST p10, with or without 25HC (10 μM) for 24 hr. The red arrows highlight the syncytia formation. Scale bar: 200 μm. Quantification of membrane fusion assays was performed by calculating the number of cells in GFP positive syncytia.

For all figures, experiments were repeated at least three times with similar results. Data are represented as mean ± SEM. Statistical significance is from pooled data of the multiple independent experiments (*$p \leq 0.05$).

Figure S4

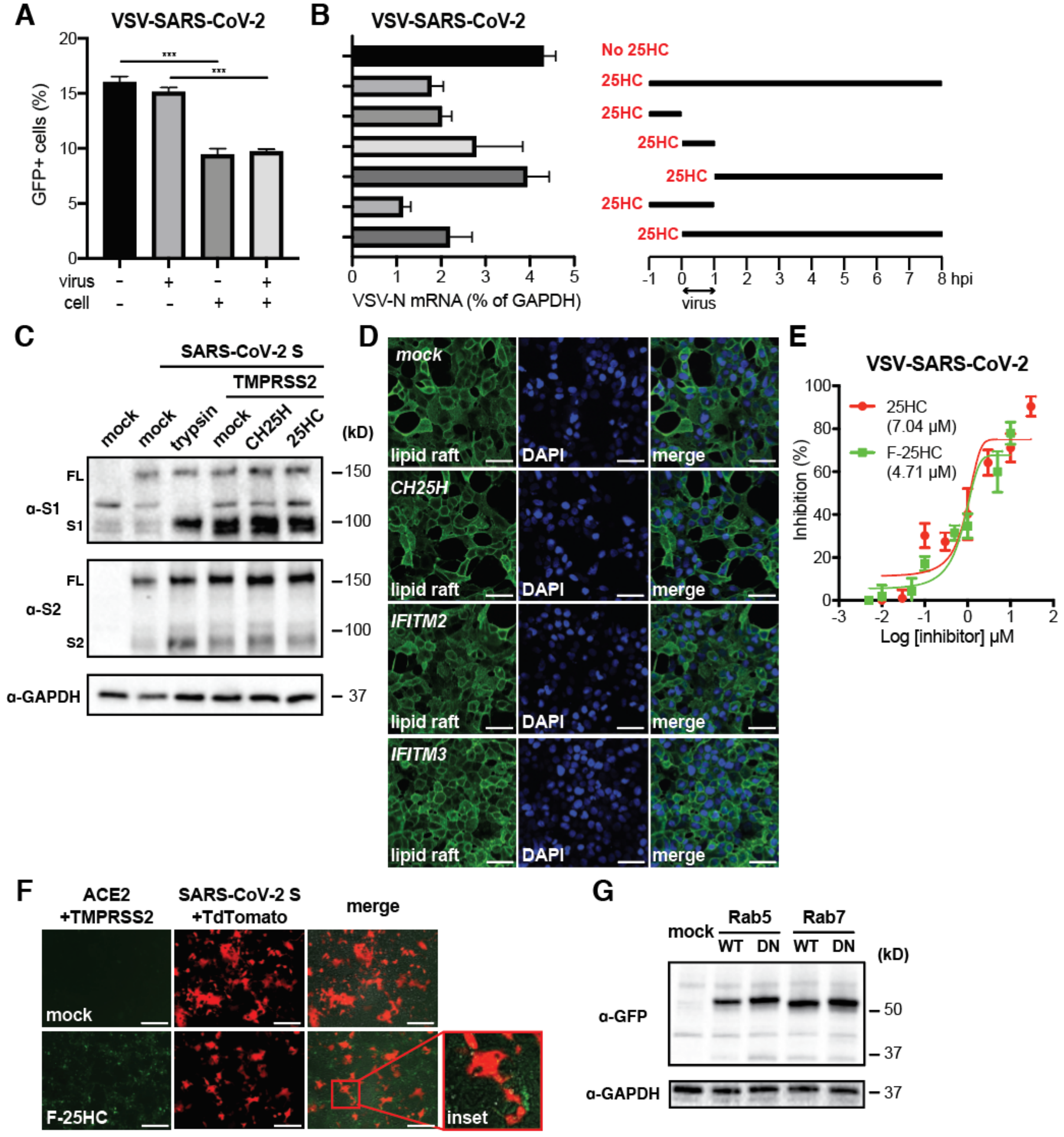

Fig. S4. CH25H and 25HC do not affect S cleavage or lipid raft organization.

(A) VSV-SARS-CoV-2 (virus) was pre-incubated with 25HC (+, 10 μM) for 30 min and used for infection. Alternatively, HEK293-hACE2 cells (cell) were treated with 25HC (+, 10 μM) for 1 hr and infected with VSV-SARS-CoV-2 (MOI=1). At 6 hpi,

cells were harvested and measured for GFP percentage and intensity by flow cytometry.

(B) MA104 cells were treated with 25HC (10 μM) based on the scheme (right panel) and infected with VSV-SARS-CoV-2 (MOI=1). At 24 hpi, the mRNA level of VSV N was measured by RT-qPCR and normalized to GAPDH expression (left panel).

(C) HEK293-hACE2 cells were transfected with SARS-CoV-2 for 24 hr with or without TMPRSS2 and CH25H transfection, trypsin (0.5 μg/ml), or 25HC (10 μM). Cells were harvested for western blot and probed for SARS-CoV-2 S1, S2, and GAPDH protein levels. FL: full-length SARS-CoV-2 S; S1, S2: S cleavage products.

(D) HEK293-hACE2 cells stably expressing indicated ISGs were stained for lipid rafts (cholera toxin B, green) and nucleus (DAPI, blue). Scale bar: 30 μm.

(E) MA104 cells were treated with 25HC (0.01-30 μM) or C4-TopFluor-25HC (0.003-10 μM) for 1 hr and infected with VSV-SARS-CoV-2 (MOI=0.5) for 24 hr. At 24 hpi, the mRNA level of VSV N was measured by RT-qPCR and normalized to GAPDH expression and plotted as percentage of inhibition.

(F) HEK293-hACE2-TMPRSS2 cells were treated with or without C4 TopFluor-25HC (F-25HC, 3 μM) and co-cultured at 1:1 ratio with HEK293 cells transfected with SARS-CoV-2 and TdTomato for 24 hr. Note that the fused cells (red) stop at the boundary of 25HC treated cells (green). Scale bar: 200 μm.

(G) HEK293-hACE2 cells were transfected GFP-tagged wild-type (WT) or dominant negative (DN) mutants of Rab5 or Rab7 for 24 hr. Cells were harvested for western blot and probed for GFP and GAPDH protein levels.

For all figures, experiments were repeated at least three times with similar results. Data are represented as mean ± SEM. Statistical significance is from pooled data of the multiple independent experiments (***$p \leq 0.001$).

Figure S5

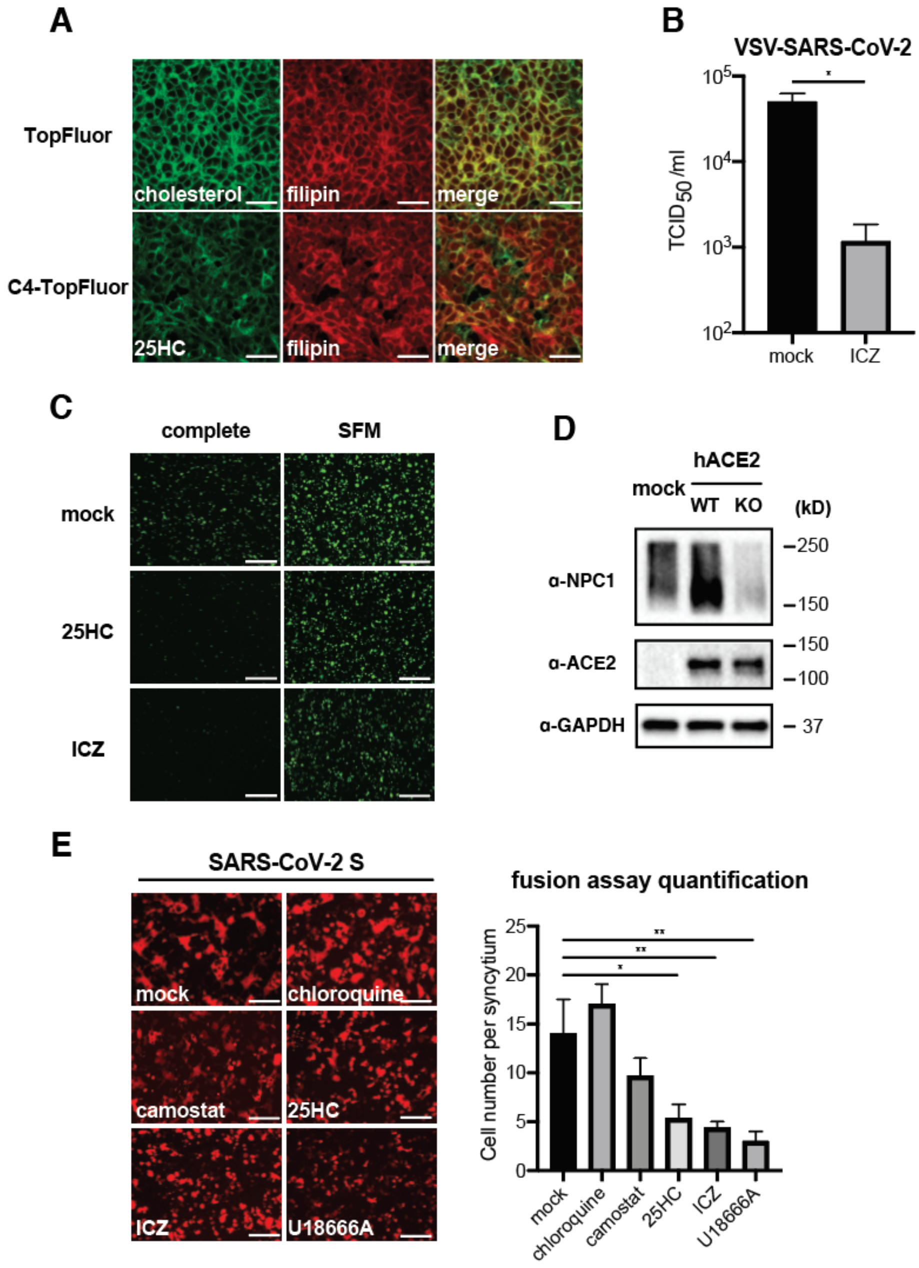

Fig. S5. Inhibition of NPC1 reduced SARS-CoV-2 fusion and infection

(A) HEK293 cells were incubated with TopFluor-cholesterol (2 μM, green) or C4-TopFluor-25HC (2 μM, green) for 1 hr and stained with 50 μg/ml filipin for 30 min (pseudo-colored in red). Scale bar: 30 μm.

(B) HEK293-hACE2 cells were treated with ICZ (3 μM) for 16 hr and infected with VSV-SARS-CoV-2 (MOI=1). At 24 hpi, cell lysates were harvested for $TCID_{50}$ assays.

(C) MA104 cells were treated with 25HC at indicated concentrations in either complete or serum-free media (SFM) for 1 hr and infected with VSV-SARS-CoV-2 (MOI=1). GFP signals at 24 hpi were captured by the fluorescence microscope.

(D) HEK293-hACE2-TMPRSS2 cells were harvested for western blot and probed for NPC1, ACE2, and GAPDH protein levels.

(E) HEK293-ACE2-TMPRSS2 cells were transfected with SARS-CoV-2 S and TdTomato plasmids for 24 hr in the presence of chloroquine (10 μM), camostat (10 μM), 25HC (20 μM), ICZ (5 μM), or U18666A (5 μM). Scale bar: 200 μm. Quantification of membrane fusion assays was performed by calculating the number of cells in TdTomato positive syncytia.

For all figures, experiments were repeated at least three times with similar results. Data are represented as mean ± SEM. Statistical significance is from pooled data of the multiple independent experiments (*$p \leq 0.05$; **$p \leq 0.01$).

Table S1. Taqman and SYBR Green QPCR primer:

SARS-CoV-2 N	Probe	6FAM/TCAAGGAACAACATTGCCAA/3BHQ
	Forward	ATGCTGCAATCGTGCTACAA
	Reverse	GACTGCCGCCTCTGCTC
rotavirus NSP5	Probe	CY5/TCAAATGCAGTTAAGACAAATGCAGACGCT/IAbRQSp
	Forward	CTGCTTCAAACGATCCACTCAC
	Reverse	TGAATCCATAGACACGCC
adenovirus 5 hexon	Forward	GACATGACTTTCGAGGTCGATCCCATGGA
	Reverse	CCGGCTGAGAAGGGTGTGCGCAGGTA
GAPDH	Forward	GGAGCGAGATCCCTCCAAAAT
	Reverse	GGCTGTTGTCATACTTCTCATGG
IFNB	Forward	ATGACCAACAAGTGTCTCCTCC
	Reverse	GGAATCCAAGCAAGTTGTAGCTC
IFNL3	Forward	TAAGAGGGCCAAAGATGCCTT
	Reverse	CTGGTCCAAGACATCCCCC
VSV N	Forward	GATAGTACCGGAGGATTGACGACTA
	Reverse	TCAAACCATCCGAGCCATTC

***CH25H* CRISPR/Cas9 single-guide RNA:**

CCTGGATATCCTGTGCTCCT

***CH25H* knockout Sanger sequencing primers:**

Forward: CCTCCTACAGTCGCCCTTCT
Reverse: CAGCGTCACGGGGAACAC

CH25H H242Q, H243Q mutagenesis primers:

Forward: GTGCACCACGACCTGCAGCAATCTCACTTTAACTGCAACT
Reverse: AGTTGCAGTTAAAGTGAGATTGCTGCAGGTCGTGGTGCAC

***NPC1* CRISPR/Cas9 single-guide RNA:**

AAAGAGTTACAATACTACGT
